# Testing the Performance of Multi-Frequency Low-Cost GNSS Receivers and Antennas

**DOI:** 10.3390/s21062029

**Published:** 2021-03-12

**Authors:** Veton Hamza, Bojan Stopar, Oskar Sterle

**Affiliations:** Faculty of Civil and Geodetic Engineering, University of Ljubljana, Jamova Cesta 2, 1000 Ljubljana, Slovenia; bojan.stopar@fgg.uni-lj.si (B.S.); oskar.sterle@fgg.uni-lj.si (O.S.)

**Keywords:** GNSS, low-cost instruments, geodetic instruments, comparison, sessions

## Abstract

Global Navigation Satellite System (GNSS) low-cost multi-frequency receivers are argued as an alternative to geodetic receivers for many applications. Calibrated low-cost antennas recently became available on the market making low-cost instruments more comparable with geodetic ones. The main goal of this research was to evaluate the noise of low-cost GNSS receivers, to compare the positioning quality from different types of low-cost antennas, and to analyze the positioning differences between low-cost and geodetic instruments. The results from a zero baseline test indicated that the u-blox multi-frequency receiver, namely, ZED-F9P, had low noise that was at the sub-millimeter level. To analyze the impact of the antennas in the obtained coordinates, a short baseline test was applied. Both tested uncalibrated antennas (Tallysman TW3882 and Survey) demonstrated satisfactory positioning performance. The Tallysman antenna was more accurate in the horizontal position determination, and the difference from the true value was only 0.1 mm; while, for the Survey antenna, the difference was 1.0 mm. For the ellipsoid height, the differences were 0.3 and 0.6 mm for the Survey and Tallysman antennas, respectively. The comparison of low-cost receivers with calibrated low-cost antennas (Survey Calibrated) and geodetic instruments proved better performance for the latter. The geodetic GNSS instruments were more accurate than the low-cost instruments, and the precision of the estimated coordinates from the geodetic network was also greater. Low-cost GNSS instruments were not at the same level as the geodetic ones; however, considering their cost, they demonstrated excellent performance that is sufficiently appropriate for various geodetic applications.

## 1. Introduction

Global Navigation Satellite System (GNSS) is a widely used technology for different positioning and navigation purposes. When the highest possible accuracy is required, then geodetic GNSS instruments are typically used in various applications [1,2,3,4,5]. The positioning quality obtained on the basis of geodetic GNSS instruments is dependent on their quality; however, to obtain the highest quality results, many errors must be accounted for. Positioning quality on the level of a few millimeters requires the biases to be strictly dealt with—they must be either modeled, mitigated, eliminated, or estimated within the least-squares process [6,7,8]. The increase in the positional quality was achieved with the development of GNSS instruments, modeling biases, processing algorithms, and also with the advent of multi-GNSS [9,10].

Geodetic GNSS instruments are able to measure code and phase pseudo-ranges with high accuracy and precision that indicates better performance [11]. However, they are argued as too expensive in certain applications and, therefore, low-cost GNSS instruments may be seen as a suitable alternative [11,12,13,14,15,16,17,18].

### 1.1. Overview

Low-cost GNSS receivers have been analyzed and tested in various applications [16,19,20,21,22,23]. Tests were typically carried out over short baselines and in favorable surveying conditions, i.e., in an open-sky environment [12,17,24]. However, in cases where their performance was tested in challenging conditions and over long baselines, the results showed a decrease in quality but were still acceptable considering their costs [15,22]. The combination of low-cost GNSS receivers with geodetic antennas has been shown to improve their performance [16].

To test the performance of GNSS receivers, only the zero baseline test or short baseline test is usually applied [25,26,27,28,29]. Within the zero baseline, all external errors are eliminated with the construction of double differences, and therefore only the receiver error remains. A study by Roberts et al. [26] analyzed the accuracy of Global Positioning System (GPS)-only, BeiDou (BDS)-only, and a combination of GPS/BDS within zero baseline, where geodetic instruments were used. The results on a basis of GPS showed better performance compared to BDS; however, the latter has not yet been fully operational.

A detailed study of code and phase observation noise for two types of geodetic receivers was performed by Zhang et al. [29]. The results showed comparable quality of the code observations, whereas, in the case of phase observations, significant differences in the quality between different receivers were obtained [29]. A study by Amiri-Simkooei and Tiberius [28] reported that the receiver noise was slightly different for several receivers from the same manufacturer. The evaluation was done with the zero baseline test and also with a short baseline test, where for the latter, harmonic functions were used to compensate for the multipath error. In the case of Dróżdż and Szpunar [30], the noise of observations was determined with a signal simulator, and the results indicated that the quality of observations may vary among different receiver types. The study by Han et al. [31] used an signal-to-noise-dependent environmental model, where observation weights are determined based on the satellite elevation angle in order to reduce the noise in harsh environments. The noise of GPS/GLONASS or GPS/BDS was lower than the noise of GPS-only, and the combination of measurements from more constellations was shown to improve the quality of the obtained coordinates [32].

Geodetic GNSS instruments were used as a reference to test low-cost instruments in dynamic and static scenarios, and the results indicated that, in certain applications, low-cost instruments can have satisfactory performance that is comparable with geodetic instruments [18,19]. Geodetic as well as single-frequency low-cost GNSS receivers were tested in Real-Time Kinematic (RTK) mode following the International Organization for Standardization (ISO) standard 17123-8. The uncertainties for the geodetic instruments were 2.5 and 4.5 mm for the horizontal and vertical components, respectively, whereas, in the case of low-cost instruments, the uncertainties were 5.5 and 11 mm for the horizontal and vertical components, respectively. However, over short baselines, low-cost instruments were comparable to the geodetic GNSS instruments [12]. In the case of Lambrou and Kanellopoulos [33], GNSS instruments were tested in virtual reference station-RTK positioning mode, where the accuracy and precision of the GNSS instruments were analyzed. The study by Sioulis et al. [17] tested u-blox NEO-7P receivers in RTK mode, where the results met the requirements defined in Class 2 of the ISO RTK testing procedure. A single-frequency EVK-M8T receiver in combination with a survey-grade antenna was tested in RTK mode with short baselines. The results indicated that the low-cost receivers achieved comparable performance with the high-ended geodetic GNSS instruments [34]. Ambiguity resolution and positioning performance in the RTK mode for the EVK-M8T receiver were compared with dual-frequency GPS geodetic receivers. The results showed that there was competitive performance with the geodetic GNSS receivers, even though small ionospheric delays were present [35]. The study by Odolinski and Teunissen [36] emphasized that single-frequency u-blox EVK-M8T receivers (L1 + B1) with patch antennas reached similar ambiguity resolution performance in RTK as double frequency geodetic receivers (L1 + L2 GPS).

Low-cost u-blox LEA 4T receivers were evaluated in a controlled error-free environment in static as well as dynamic scenarios [25]. They showed that the level of multipath was much smaller in a static scenario; however, in the case of a dynamic scenario, the multipath may severely degrade the performance. U-blox LEA-6T and NEO-7P receivers were tested with geodetic antennas over short and long baselines in static mode by following the guidelines that consisted of the ISO standards. Both receivers showed low receiver noise and baseline vectors, and the coordinates were determined with accuracy comparable to the geodetic receivers with short baselines. In long baselines, the low-cost receivers showed worse results, but were still satisfactory [18]. The study by Guo et al. [11] reported that low-cost single-frequency receivers provided low noise in open sky conditions, and their performance was comparable with geodetic receivers over short baselines.

Recently, multi-frequency low-cost GNSS receivers were released on the market that can track signals in both L1 and L2 frequencies from all available constellations. Nie et al. [37] proposed a new method where single-frequency ionosphere-corrected code observations, dual-frequency ionospheric-free code and phase observations, and precise ionospheric products are combined in one model for dual-frequency low-cost (ZED-F9P) receivers. The results indicated that the proposed method reached a faster horizontal accuracy of half a meter in real-time precise point positioning. Dual-frequency receiver (ZED-F9P) in combination with a u-blox ANN-MB patch antenna was tested in short and long-baseline in RTK mode. In long baselines, it had worse performance compared to the geodetic GNSS instruments [38]. Multi-frequency low-cost GNSS receivers were also evaluated in combination with geodetic as well as low-cost antennas [14,19,20,39]. Lastly, calibrated low-cost antennas are available in the market, which makes multi-frequency low-cost receivers even more comparable with geodetic GNSS instruments.

### 1.2. Work Organization

In this study, we evaluated the performance of a multi-frequency low-cost receiver, namely, ZED-F9P, in combination with different types of low-cost antennas. The main objectives were to analyze the noise of the receiver, to evaluate its positioning performance with different types of low-cost calibrated and non-calibrated antennas, and to compare the quality of the coordinates obtained from geodetic and low-cost GNSS instruments.

First, the procedure of testing the GNSS receiver positional quality is described, with a special focus on the performance of the low-cost receivers (Section 1). The implemented zero baseline test, short baseline test with different types of low-cost antennas, and the comparison of coordinates obtained from low-cost and geodetic GNSS instruments are shown in Section 2. The results are presented and discussed in Section 3. In the end, the conclusions are listed (Section 4).

## 2. Materials and Methods

The rooftop of the Faculty of Civil and Geodetic Engineering, University of Ljubljana (UL FGG) building was chosen as the location of all tests. This is an open area where less multipath error is expected, and three pillars with force centering systems marked as FGG1, FGG2, and FGG4 were established. The coordinates of these pillars are well known from several long static GNSS surveys, performed in the past. These points were, therefore, chosen for the purposes of our study.

The SimpleRTK2B V1 board (170 EUR), which is offered by Ardusimple manufacturer (Lleida, Spain), housed the u-blox ZED-F9P low-cost chip that can receive satellite signals (L1C/A, L1OF, E1, B1l, L2C, L2OF, E5b, and B2l) in both frequencies from GPS, GLONASS, BDS, and Galileo constellations [40]. The board is compatible with different types of low-cost antennas and was used in all tests. The Survey GNSS multiband antenna (90 EUR) from Ardusimple manufacturer and Tallysman TW 3882 (Ottawa, Canada) (290 EUR) used in this research are non-calibrated but are reported to have tight phase center variations [41,42]. On the other side, recently, low-cost calibrated antennas, such as the Survey Calibrated GNSS multiband antenna (149 EUR), are available in the market and can be more comparable with geodetic ones [41].

### 2.1. Zero Baseline Test

**Figure 1:**
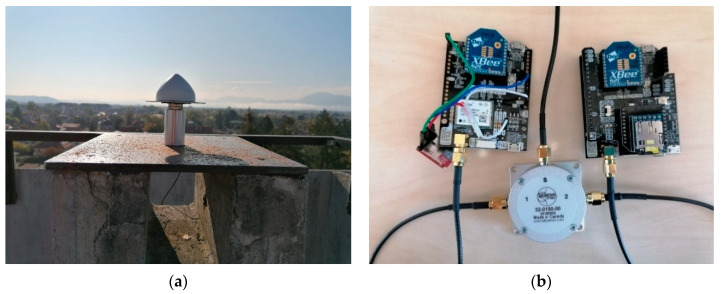
Global Navigation Satellite System (GNSS) instruments used in the zero baseline test: (**a**) Tallysman antenna TW3882; (**b**) Signal splitter TW 150 L and SimpleRTK2B V1 boards.

The zero baseline test was used to estimate the noise of both receivers since all other errors are eliminated by sharing the same signal from a single antenna [18]. For this test, we used dual-frequency receivers (ZED-F9P) in combination with a low-cost antenna (Tallysman TW 3882); one TW 150 L Band/GNSS 1 to 2 signal splitter was used to split the signals from the antenna and redirect it to both receivers (Figure 1).

The antenna was placed on point FGG4 and observations were carried out for 24 h at a rate of 1 Hz. The GNSS data were processed with the open-source software RTKLIB (demo5_b33b), and the adopted parameters are shown in Table 1 [43]. These parameters are used for all processing, where only the elevation mask or duration may change from one case to another.

Since all errors are eliminated by sharing the same antenna, the remaining error is referred to as the combined noise of both receivers. Assuming that both receivers have the same performance and considering the variance-covariance propagation law, the noise (uncertainty in coordinates) is estimated as follows:

(1) $$\sigma_{r}=\sigma_{t}/\sqrt{2},$$

where $\sigma_{t}$ is the total noise from both receivers, and $\sigma_{r}$ is the noise of the single receiver.

Within the first analysis, double-difference phase observations were processed in kinematic mode for the zero-baseline to obtain the kinematic components of the zero-baseline for each epoch. The estimated baseline components are shown for the east-*e*, north-*n*, and height-*h* components. To analyze the performance of both low-cost receivers of the zero-baseline, both stations were then used as rover stations in the second analysis, where the permanent station GSR1 was selected as the base station.

GSR1 is located 3.5 km away from the UL FGG and is one of the permanent stations that form the national Slovenian network of permanent GNSS stations. This station is equipped with the LEIAT504GG choke ring antenna and LEICA GRX1200GGPRO receiver [44]. Observations were carried out for 10 h at 1 Hz. For the point FGG4, two triplets of coordinates were estimated, both from low-cost receivers. The first set is denoted as $e_{A}$, $n_{A}$, and $h_{A}$, while the second one is denoted as $e_{B}$, $n_{B}$, and $h_{B}$. The differences between the 1*D*, 2*D*, and 3*D* positions are calculated as:

(2) $$d_{1D}=h_{A}-h_{B},$$

(3) $$d_{2D}=\sqrt{{\left(e_{A}-e_{B}\right)}^{2}+{\left(n_{A}-n_{B}\right)}^{2}},$$

(4) $$d_{3D}=\sqrt{{\left(e_{A}-e_{B}\right)}^{2}+{\left(n_{A}-n_{B}\right)}^{2}+{\left(h_{A}-h_{B}\right)}^{2}}.$$

### 2.2. Short Baseline Test

The short baseline test was used to evaluate different low-cost GNSS antennas since most external errors were eliminated. Three antennas were selected, namely, the Survey, the Tallysman, and the Survey Calibrated antenna. While the Survey and Tallysman antennas are not calibrated, the phase center variation of the Survey Calibrated antenna is known [41,42]. For the performance analysis, four baselines were defined. The first and second baseline consisted of uncalibrated low-cost antennas (Survey–Survey and Tallysman–Tallysman), the third one consisted of two calibrated low-cost antennas (Survey calibrated–Survey calibrated), while the last one was observed with the geodetic GNSS instrument Leica GS18.

**Figure 2:**
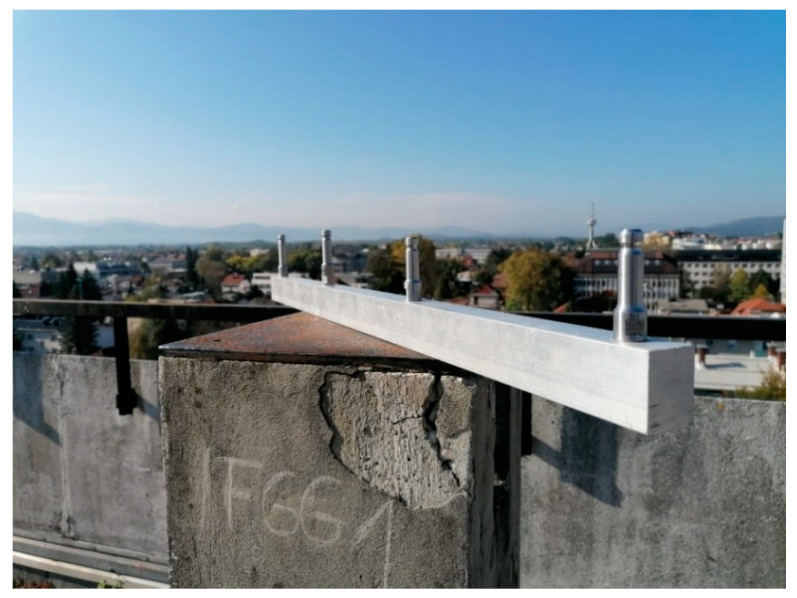
The special four-point metal arm used in the short baseline test.

For the short baseline test, a special four-point metal arm was constructed to measure the GNSS observations with four GNSS antennas simultaneously. The inner-geometry of the four-point metal arm was determined with high accuracy (0.05 mm), the horizontal distances between consecutive points were all 25.0 cm, while special metal mechanisms were used to ensure the same height for all antennas (Figure 2).

#### 2.2.1. Comparison of Low-Cost Uncalibrated Antennas

Observations were acquired at the same location with the Tallysman and Survey antennas at 1 Hz for 50 h and the data were processed in 40-min sessions. The processing was done with a 20 min gap between the sessions to ensure changes in the satellite constellation geometry. For each session, the horizontal coordinates e and n and the ellipsoid height h were estimated.

**Figure 3:**
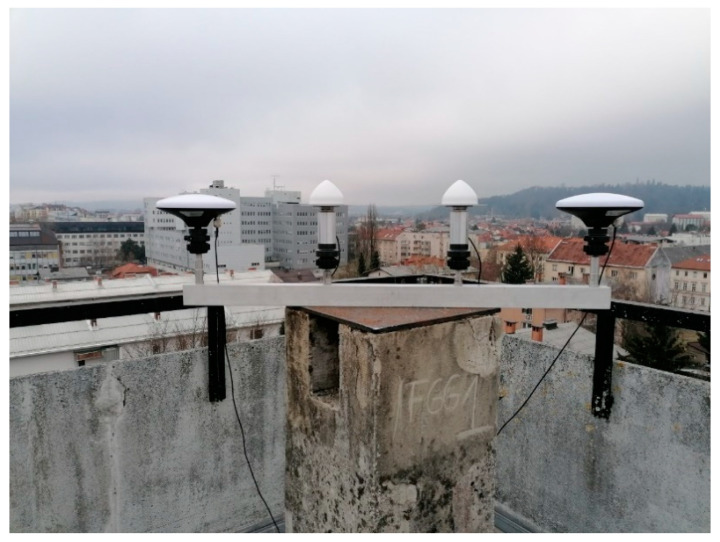
Low-cost uncalibrated antennas with the 4-point metal arm.

The differences in the estimated coordinates are due to the impact of the antennas, since the same type of receivers were used to observe at the same location (Figure 3). The estimated coordinates were then used to calculate the horizontal distances and ellipsoid heights. To analyze the distances and ellipsoid heights, certain elementary statistics, such as the minimum and maximum values of the residuals and root-mean-square-error (RMSE) were computed. For the outlier detection, a τ-test was applied [45]. To compare the estimated horizontal distances and height components with their true values, the T-test was used, and the following hypotheses were defined:

*H*_0_: The estimated horizontal distance is equal to the true horizontal distance.

*H_a_*: The estimated horizontal distance is not equal to the true horizontal distance.

Similarly, the hypothesis for the ellipsoid height were defined as:

*H*_0_: The estimated ellipsoid height is equal to the true ellipsoid height.

*H_a_*: The estimated ellipsoid height is not equal to the true ellipsoid height.

#### 2.2.2. Comparison of Geodetic and Low-Cost GNSS Instruments

**Figure 4:**
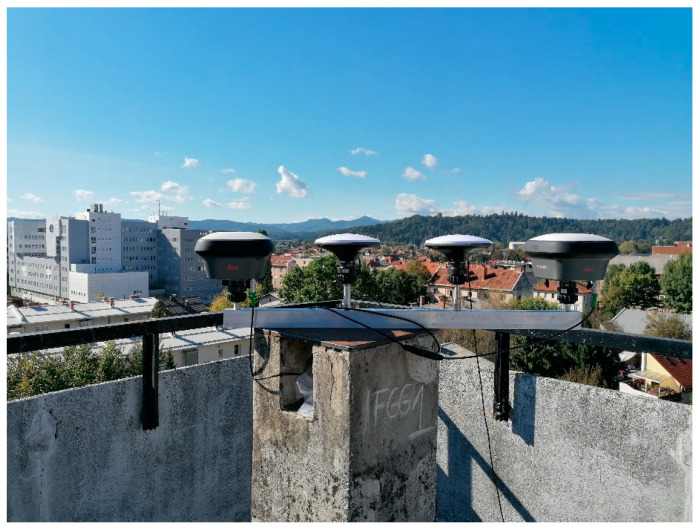
Geodetic and low-cost calibrated antennas with the four-point metal arm.

We obtained horizontal distances and ellipsoid heights from the estimated coordinates that were determined from low-cost (ZED-F9P receiver and Survey Calibrated antenna) and geodetic GNSS instruments (Leica GS18 receiver and LEIGS18 antenna). To ensure the same conditions as in the previous case (see Section 2.2.1), 50 h of GNSS data with a 1 Hz sampling rate were acquired (Figure 4). Similar statistics as in the previous case were obtained to analyze the results. To compare the horizontal distance from both types of instruments, the analysis of variance (ANOVA) was used with the following hypotheses [46]:

*H*_0_: The use of different GNSS instruments does not influence the horizontal position.

*H_a_*: The use of different GNSS instruments influences the horizontal position.

The same analysis was done for the vertical component where the hypotheses were set as:

*H*_0_: The use of different GNSS instruments does not influence the ellipsoid height.

*H_a_*: The use of different GNSS instruments influences the ellipsoid height.

The results from this comparison are presented in Section 3.2.2.

### 2.3. Comparison of Coordinates from Geodetic Network

**Figure 5:**
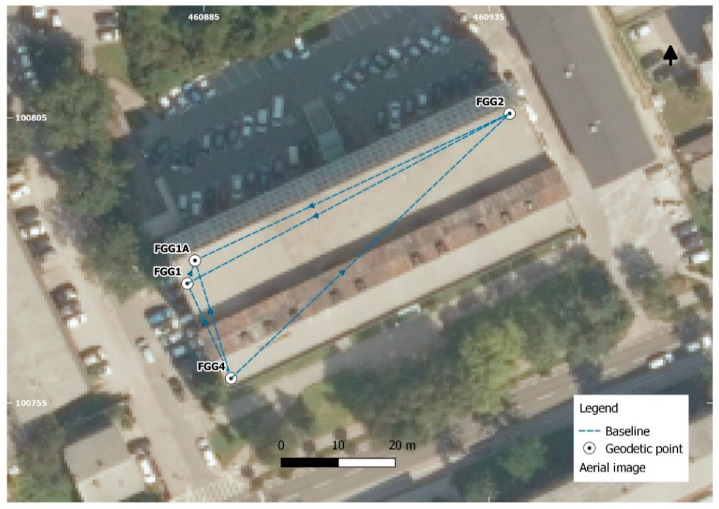
The geodetic network.

Low-cost GNSS receivers have been seen as an alternative solution to geodetic ones, particularly in cases where a risk for instrument damage (natural hazards) is present and the highest accuracy is not required. To analyze if low-cost receivers are able to provide coordinates with comparable precision to the geodetic receivers, a geodetic network consisting of four points was established (Figure 5).

The same network was observed with the above-mentioned (Section 2.2.2) geodetic GNSS instruments (first session) and low-cost GNSS instruments (second session) for 3 h at 1 Hz. The FGG4 point was chosen as a datum point while the misclosure of the baseline vectors in triangles before the adjustment was obtained. The minimally constrained adjustment of the baseline vectors in the network was performed for both sessions and the a posteriori variance was estimated [47]. In the case that both GNSS instruments can ensure the same network precision, the a posteriori variances need to be statistically equal. The equality of both variances was tested on based on the following hypotheses [45]:

$$H_{0}=E\left({\hat{\sigma }}_{G}^{2}\right)=E\left({\hat{\sigma }}_{L}^{2}\right),$$

$$H_{a}=E\left({\hat{\sigma }}_{G}^{2}\right)\neq E\left({\hat{\sigma }}_{L}^{2}\right),$$

where ${\hat{\sigma }}_{G}$ is a posteriori variance from geodetic GNSS instruments, and ${\hat{\sigma }}_{L}$ is the a posteriori variance from low-cost GNSS instruments.

Verification of the rejection of the null hypothesis was performed by the following test, which belongs to the *F*-distribution [45]:

(5) $$F=\frac{{\hat{\sigma }}_{G}^{2}}{{\hat{\sigma }}_{L}^{2}}\leq F_{1-\alpha ,f_{1},f_{2}},$$

(6) $$f_{1}=n_{1}-u_{1}+d,$$

(7) $$f_{2}=n_{2}-u_{2}+d,$$

where *f*_i_ is the degrees of freedom for a certain session, *n*_i_ is the number of observations for certain session, *u*_i_ is the number of unknowns for a certain session, *d* is the datum defect, and *α* is the significance level.

The comparison of the estimated coordinate precision can be done using the *F*-test Equation (5) in the case where the null hypothesis is not rejected, and homogenous precision is achieved in both sessions.

## 3. Results and Discussion

In this section, the results from the previous sections are presented. The results from the zero baseline test are presented in Section 3.1, the results from the short baseline test are in Section 3.2, and the results from the coordinate comparison within the geodetic network are in Section 3.3.

### 3.1. Zero Baseline Test Results

**Figure 6:**
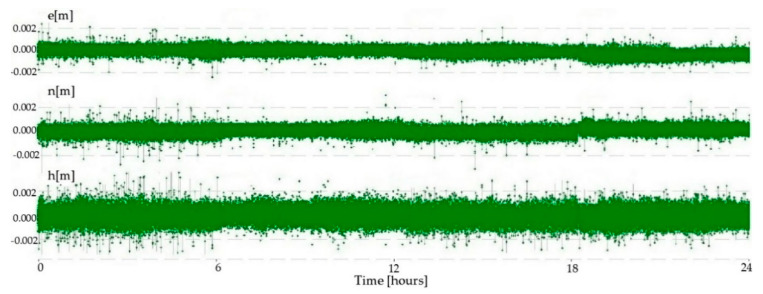
The zero baseline test position errors for the east (*e*), north (*n*), and ellipsoid height (*h*).

Multi-frequency low-cost GNSS receivers have been shown to have low noise of phase observations. They can measure the phase observations with high precision, which resulted in small errors of the baseline components, and the estimated noise was on the sub-millimeter level (Table 2). Kinematic processing showed that the position error was in the interval of ±1 mm for both horizontal components, while it was in the interval of ±2 mm for the vertical component (Figure 6).

To estimate the differences in the position determination, the same low-cost antenna and two low-cost receivers set at the point FGG4 and a geodetic receiver with a geodetic antenna (permanent station GSR1) as a reference station were used. The coordinate differences were at the millimeter level for the ellipsoid height and at the sub-millimeter level for the horizontal components. The obtained differences in position were 0.9, 1.0, and 1.4 mm for the 2*D* position (horizontal), 1*D* position (vertical), and 3*D* position, respectively (Table 3). The results showed that the performance of both low-cost receivers was comparable and of high precision.

### 3.2. Short Baseline Test Results

#### 3.2.1. Results from the Comparison of the Low-Cost Uncalibrated Antennas

The low-cost uncalibrated antennas Survey and Tallysman were tested on a four-point metal arm with known inner-geometry and in an open sky area where small multipath error was expected. Since the GNSS antennas were placed only 25.0 cm and the same GNSS receivers were used apart, the majority of the GNSS biases were eliminated by forming phase single differences.

**Figure 7:**
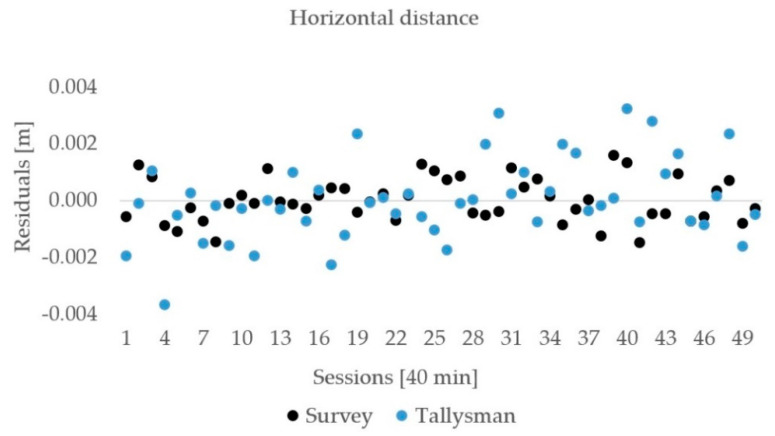
Horizontal distance residuals for the low-cost uncalibrated antennas.

**Figure 8:**
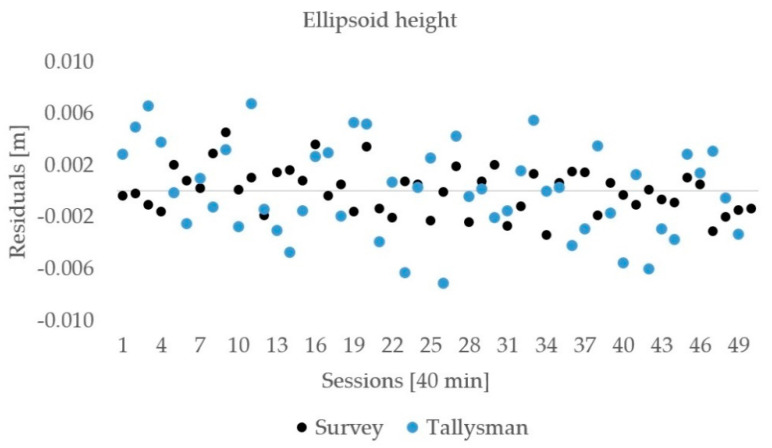
Ellipsoid height residuals for the low-cost uncalibrated antennas.

The horizontal distances between the antennas and ellipsoid heights of the rover were estimated 50 times, and each of them was calculated from the GNSS observations that lasted for 40 min. The distribution of the residuals with respect to the mean are shown for the horizontal distance in Figure 7 and for the ellipsoid height in Figure 8. The estimated horizontal distances and ellipsoid heights were in intervals of less than 3σ, and no outliers were detected. Elementary statistics are presented in Table 4 and Table 5 for the horizontal distance and ellipsoid height, respectively.

The differences of the mean (averaged) horizontal distance from the true value were −0.1 mm for the Tallysman antenna and −1.0 mm for the Survey antenna. The RMSE values of the horizontal distance were 1.4 and 0.8 mm for the Tallysman and Survey antenna, respectively. For the ellipsoid height, the RMSE values were 3.9 mm for the Tallysman and 1.7 mm for the Survey antenna. The difference from the true value was at the sub-millimeter level for both of them. Based on the results, we may conclude that both antennas performed well and provided millimeter precision for the horizontal distance and a few millimeters for the ellipsoid height.

The T-test was performed to compare the estimated distance and ellipsoid height difference to their true values (Table 6). The results showed that we are not able to reject the null hypothesis with a significance level of 5% for both horizontal distances as well as for the ellipsoidal height in the case of the Tallysman antenna; however, in the case of the Survey antenna, we were not able to reject the null hypothesis in the case of the height component; however, we can reject the null hypothesis for horizontal distances. This is due to the difference of the horizontal distance from the expected value that is −1.0 mm for the Survey antenna and only −0.1 for the Tallysman.

#### 3.2.2. Results from the Comparison of Geodetic and Low-Cost GNSS Instruments

Static observations were performed with geodetic (Leica GS18 receiver and LEIGS18 antenna) and low-cost (ZED-F9P receiver and Survey Calibrated antenna) GNSS instruments at the same time using a four-point metal arm to ensure equal conditions since different types of equipment were used.

**Figure 9:**
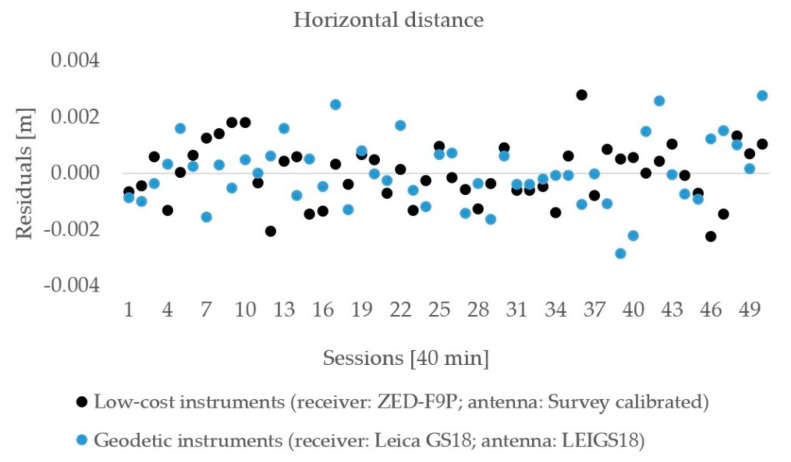
The horizontal distance residuals for geodetic and low-cost GNSS instruments.

**Figure 10:**
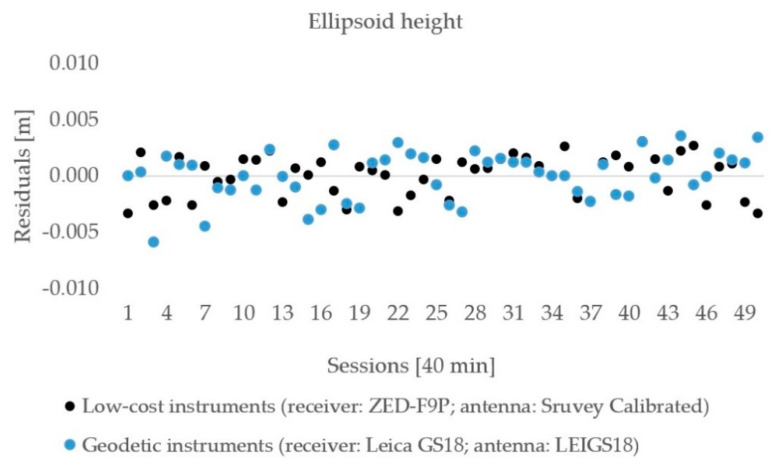
The ellipsoid height residuals for geodetic and low-cost GNSS instruments.

The observations lasted for 50 h and were processed in 40 min sessions with 20 min gaps among them to allow for changes in the satellite distribution geometry. The residuals of the horizontal distances are shown in Figure 9, and the ellipsoid heights are presented in Figure 10. No outliers were detected in the estimated horizontal distances and ellipsoid heights. The above–mentioned statistics (Section 2.2.1) are presented in Table 7 and Table 8 for both components.

The residuals of the horizontal distances for both the geodetic and low-cost instruments were in an interval of ±3 mm according to the mean values. The RMSE of the low-cost GNSS equipment was 1.0 mm while, for the geodetic GNSS instruments, was 1.2 mm; however, the difference of the horizontal distance to its true value was smaller in the case of geodetic equipment (0.3 mm) compared with for low-cost equipment at −1.2 mm.

The vertical component was less accurate compared with the horizontal one, which was confirmed for both used equipment. The residuals were in an interval of ±6 mm for geodetic and low-cost instruments. The RMSE of ellipsoid height obtained from the low-cost instruments was 1.8 mm, while it was 2.1 mm for the geodetic one. Geodetic instruments showed a slightly smaller difference of the ellipsoid height from the true (0.7 mm) rather than low-cost instruments (0.8 mm).

Table 9 represents the results from the ANOVA test, which was performed with a significance level of 5%. Based on the obtained results, the null hypothesis (Section 2.2.2) was rejected for both components, and the use of different types of GNSS equipment affected the horizontal position and ellipsoid height.

### 3.3. Coordinate Comparison Results (Geodetic Network)

The geodetic network that consisted of four points was used to compare the coordinates from the different types of GNSS instruments. The coordinates were obtained on a basis of 3 h of static GNSS data for both sessions. In total, six triangles were defined, and the misclosures of the triangles are presented in Table 10 and Table 11 for the geodetic (first session) and for low-cost (second session) receivers, respectively. The results indicate that the misclosures of the triangles in the case of geodetic receivers are at the level of 1–2 mm, while the misclosures of the triangles in the case of low-cost receivers are greater and reach up to 6.5 mm.

The geodetic network adjustment exposed the difference in the determined a posteriori variance between both GNSS instruments. The null hypothesis defined in Section 2.3 was rejected, and therefore inhomogeneous precision was achieved for both networks. Geodetic receivers provide a standard deviation of 0.4 mm, which was four–times better than the standard deviation of 1.8 mm determined with the low-cost instruments (Table 12).

The results from Table 12 show that the geodetic baseline components are determined with 0.4 mm precision, whereas the low-cost baseline components had a couple of millimeters precision. The estimated coordinates of the geodetic network are therefore determined with inhomogenous precision, and consequently no statistical comparison may be done. The differences between the estimated coordinates between both sessions (e.g., geodetic and low-cost) (*e*, *n*, and *h*) are shown in Table 13.

## 4. Conclusions

Low-cost GNSS instruments have been seen as an alternative to geodetic instruments for monitoring, positioning, and navigation purposes. The last generation of these receivers can receive satellites signal on two frequencies, and this makes them more comparable with high–ended geodetic receivers.

In this work, the noise of low-cost receivers was estimated, and the impact of different antennas on the positional quality was analyzed. Low-cost receivers with calibrated antennas were compared with geodetic instruments and the differences were discussed and analyzed. The results of this work led to the following conclusions:

- Low-cost multi–frequency GNSS receiver (ZED–F9P) had small receiver noise, which was at the sub–millimeter level.
- The tested uncalibrated antennas (Tallysman and Survey) demonstrated similar performances—the difference of the estimated horizontal and vertical components from the true value was at the millimeter level or even smaller.
- The short baseline test showed that low-cost GNSS instruments (with Survey Calibrated antennas) had similar positioning precision with the geodetic GNSS instruments; however, the latter had higher accuracy.
- Low-cost GNSS instruments can provide coordinates with a few millimeters of precision over a short time interval that is adequate in certain applications; however, they are not on the same level as geodetic instruments, considering that the obtained precision from the minimum constrain adjustment of the established geodetic network was four times better for geodetic instruments.

All the tests were performed in the open sky and in an area with small multipath error that was in the favor of the low-cost antennas, which are more sensitive to multipath. To fully evaluate the performance of the low-cost receivers, more tests will be realized in the future over long baselines and real environmental conditions where different factors, such as multipath, weather conditions, and others, can influence the results.

## Figures and Tables

**Table 1:** The parameters used in the zero baseline test.

Parameters	RTKLIB
Observations	L1, L2
Duration	24 h
Constellations	GPS, GLONASS, Galileo
Troposphere	Saastamoinen
Ambiguity	Fix and hold (LAMBDA)
Elevation mask	0°

**Table 2:** Baseline components from the zero baseline test.

Parameters	ΔX (m)	ΔY (m)	ΔY (m)	$\sigma_{r}$ (m)
Noise	−0.0001	0.0000	0.0001	0.0001

**Table 3:** The east (*e*), north (*n*), height (*h*), 1*D*, 2*D*, and 3*D* position differences.

Parameters	*e* (m)	*n* (m)	*h* (m)	1*D* (m)	2*D* (m)	3*D* (m)
Differences	0.0004	0.0008	0.0010	0.0010	0.0009	0.0014
Differences precision	0.0003	0.0000	0.0008	0.0008	0.0003	0.0009

**Table 4:** Horizontal distance statistics for the low-cost uncalibrated antennas.

Statistics	Min (mm)	Max (mm)	RMSE (mm)	Difference from True (mm)
Tallysman	−3.7	3.2	1.4	−0.1
Survey	−1.5	1.6	0.8	−1.0

**Table 5:** Ellipsoid height statistics for the low-cost uncalibrated antennas.

Statistics	Min (mm)	Max (mm)	RMSE (mm)	Difference from True (mm)
Tallysman	−6.3	6.8	3.9	−0.6
Survey	−3.4	4.5	1.7	0.3

**Table 6:** The Results from the T-test.

T-Test	Tallysman		Survey	
	Horizontal Distance	Ellipsoid Height	Horizontal Distance	Ellipsoid Height
T	0.70	1.11	9.02	1.20
T_critical_	2.01	2.01	2.01	2.01

**Table 7:** The horizontal distance statistics for low-cost instruments (*receiver*: ZED-F9P; *antenna*: Survey Calibrated) and geodetic instruments (*receiver*: Leica GS18; *antenna*: LEIGS18).

Statistics	Min (mm)	Max (mm)	RMSE (mm)	Difference from True (mm)
Low-cost	−2.3	2.8	1.0	−1.2
Geodetic	−2.9	2.7	1.2	0.3

**Table 8:** The ellipsoid height statistics for low-cost instruments (*receiver*: ZED–F9P; *antenna*: Survey Calibrated) and geodetic instruments (*receiver*: Leica GS18; *antenna*: LEIGS18).

Statistics	Min (mm)	Max (mm)	RMSE (mm)	Difference from True (mm)
Low-cost	−3.3	3.1	1.8	0.8
Geodetic	−5.9	3.5	2.1	0.7

**Table 9:** Comparison of the horizontal distance and ellipsoid height with the ANOVA statistical test.

ANOVA	Horizontal Distance	Ellipsoid Height
F	43.15	13.40
F_critical_	3.94	3.94

**Table 10:** Misclosures of triangles for the geodetic GNSS instruments.

Triangle	Misclosure		
	X (m)	Y (m)	Z (m)
FGG4-FGG1A-FGG1	−0.0012	−0.0004	−0.0004
FGG4-FGG1A-FGG2	0.0018	0.0008	0.0013
FGG2-FGG1A-FGG1	−0.0003	0.0008	0.0009
FGG2-FGG1-FGG4	0.0009	−0.0004	0.0003

**Table 11:** Misclosures of triangles for the low-cost GNSS instruments.

Triangle	Misclosure		
	X (m)	Y (m)	Z (m)
FGG4-FGG1A-FGG1	−0.0018	−0.0001	−0.0048
FGG4-FGG1A-FGG2	−0.0031	0.0010	−0.0010
FGG2-FGG1A-FGG1	0.0016	0.0010	−0.0016
FGG2-FGG1-FGG4	0.0065	−0.0019	−0.0042

**Table 12:** The geodetic network precision for both sessions: *G*—geodetic instrument and *L*—low-cost instruments.

Parameters	${\hat{\sigma }}_{G}$ (mm)	${\hat{\sigma }}_{L}$ (mm)	F_critical_	F
	0.4	1.8	2.69	17.28

**Table 13:** The coordinate differences.

Point	Δ*e* (mm)	Δ*n* (mm)	Δ*h* (mm)
FGG1	−0.7	2.3	2.9
FGG2	−1.9	1.4	0.1
FGG1A	−0.0	2.3	0.6

## Data Availability

Publicly available datasets were analyzed in this study. This data can be found here: https://unilj-my.sharepoint.com/:f:/g/personal/vhamza_fgg_uni-lj_si/EoiJ15wUVntNsBq2stpWrG4BVwO0Vs_mY6yTmb82nTAMbA?e=q0oZLd (accessed on 12 March 2021).
